# A Robust Approach to Multimodal Deepfake Detection

**DOI:** 10.3390/jimaging9060122

**Published:** 2023-06-19

**Authors:** Davide Salvi, Honggu Liu, Sara Mandelli, Paolo Bestagini, Wenbo Zhou, Weiming Zhang, Stefano Tubaro

**Affiliations:** 1 Dipartimento di Elettronica, Informazione e Bioingegneria, Politecnico di Milano, 20133 Milan, Italy; sara.mandelli@polimi.it (S.M.); stefano.tubaro@polimi.it (S.T.); 2 School of Cyber Science and Technology, University of Science and Technology of China, Hefei 230026, China; lhg9754@mail.ustc.edu.cn (H.L.); welbeckz@ustc.edu.cn (W.Z.); zhangwm@ustc.edu.cn (W.Z.)

**Keywords:** deepfake detection, video forensics, audio forensics, multimodality

## Abstract

The widespread use of deep learning techniques for creating realistic synthetic media, commonly known as deepfakes, poses a significant threat to individuals, organizations, and society. As the malicious use of these data could lead to unpleasant situations, it is becoming crucial to distinguish between authentic and fake media. Nonetheless, though deepfake generation systems can create convincing images and audio, they may struggle to maintain consistency across different data modalities, such as producing a realistic video sequence where both visual frames and speech are fake and consistent one with the other. Moreover, these systems may not accurately reproduce semantic and timely accurate aspects. All these elements can be exploited to perform a robust detection of fake content. In this paper, we propose a novel approach for detecting deepfake video sequences by leveraging data multimodality. Our method extracts audio-visual features from the input video over time and analyzes them using time-aware neural networks. We exploit both the video and audio modalities to leverage the inconsistencies between and within them, enhancing the final detection performance. The peculiarity of the proposed method is that we never train on multimodal deepfake data, but on disjoint monomodal datasets which contain visual-only or audio-only deepfakes. This frees us from leveraging multimodal datasets during training, which is desirable given their lack in the literature. Moreover, at test time, it allows to evaluate the robustness of our proposed detector on unseen multimodal deepfakes. We test different fusion techniques between data modalities and investigate which one leads to more robust predictions by the developed detectors. Our results indicate that a multimodal approach is more effective than a monomodal one, even if trained on disjoint monomodal datasets.

## 1. Introduction

Recent advances in deep learning and new media technologies have made the creation and sharing of multimedia content more accessible than ever. Users can now generate super realistic synthetic images, videos and speech tracks with minimal effort and without requiring any particular skill. The growth of these technologies can have a twofold effect. On one side, such techniques allow consumers to explore new creative and artistic possibilities and introduce applications that make everyday life easier. On the other hand, they can also lead to dangers and threats when misused. An example of the latter case are deepfakes, synthetic multimedia content generated through deep learning techniques that depict individuals in actions and behaviors that are not their own.

Deepfakes have already been used for several malicious purposes, including the publication of fabricated results in scientific journals [1] or the attack of the identity tests used by banks through synthetic voices [2] and videos [3], raising concerns about them and their use. In response to this phenomenon, the research community has prioritized the development of algorithms to discriminate real content from deepfakes [4]. Several approaches have been proposed and multiple deepfake databases have been created to push the research in this direction. Since deepfake technologies continue to advance and produce more realistic results, developing detection methods based on diverse strategies and operating principles is crucial to combat this issue.

Focusing on the analysis of video sequences, the scientific community has put forward methods for detecting deepfakes by analyzing both their audio and visual contents, as the deepfake phenomenon has impacted each of these [5]. However, while the developed detectors can demonstrate impressive performance in controlled environments, their effectiveness is somehow limited in other scenarios. For instance, most of the classifiers are monomodal, meaning that they take into account only one data modality (i.e., either visual or audio) at a time, which makes them ineffective against certain types of deepfake videos.

Visual-only detectors, for example, can be deceived by audio deepfakes, while audio-only detectors are vulnerable to deepfakes that manipulate visual content [6]. Furthermore, some information is lost during these analyses, such as the consistency between modalities, which is sometimes crucial for detecting synthetic content. To overcome these limitations, multimodal approaches have been recently proposed, able to combine information from various domains to enhance the accuracy of the detection process [7,8].

Despite their excellent performances, even multimodal methods are not immune to the problem of robustness. This refers to the ability of the detector to maintain high accuracy also when processing new unseen data, different from those used in training. This aspect is crucial in multimedia forensics, as it improves the applications of the developed systems in real-world scenarios. To address the robustness issue, researchers have explored several aspects, such as considering detectors based on different approaches and using a variety of datasets in training.

For instance, there exists a set of detectors known as semantic, which base their predictions on high-level aspects of the media under analysis [9,10]. The rationale behind these methods is that deepfake generators can reproduce low-level features but struggle with more complex aspects, making it possible to differentiate between real and synthetic data. Furthermore, these high-level features are less subject to post-processing operations applied to the data and domain changes, allowing for more robust and reliable predictions.

Regarding the use of different training datasets, it helps the developed detector not to overfit a single data type but to generalize as much as possible, improving the robustness of the final model. However, in the current literature, it is common practice to train and test the developed detectors on subsets of data extracted from the same dataset [11]. This practice can be deceptive since the high performance achieved may not be reflected when the methods are tested on different datasets. Cross-dataset tests are needed to assess the actual discrimination capabilities of the detectors.

Moreover, all the currently proposed multimodal detectors have been trained on multimodal datasets, thus requiring the presence of data of this type during the training phase. This poses an additional challenge since there is a lack of multimodal deepfake datasets proposed in the literature, while monomodal ones are widely available. For instance, the literature reports several deepfake audio datasets not including any visual content. Deepfake video datasets are available as well, though the audio tracks related to the synthetic video sequences are often taken from original speech.

In this paper, we present a new multimodal video deepfake detection method that combines visual and audio information. To determine the authenticity of the input video sequence, we combine a set of data-driven features extracted from the visual content with a set of speaker-identity features extracted from the audio content.

The peculiarity of the proposed detector is that its training phase does not take place on multimodal deepfake data but on monomodal samples. In other words, we never train our detector over video sequences that contain fully-synthetic data, i.e., where both visual and audio contents are deepfakes. During the training phase, we combine the features derived from synthetic audio and synthetic visual data extracted from disjoint monomodal datasets, meaning that we do not require any additional material with respect to training standard monomodal detectors.

We evaluate the performance of our method on several state-of-the-art multimodal video deepfake datasets by considering various fusion strategies between the two modalities. Our results show that a multimodal approach is equally more functional and robust than a monomodal one. The results show the effectiveness and robustness of the proposed approach, indicating high generalization capabilities on unseen data.

The rest of the paper is structured as follows. Section 2 provides the reader with some knowledge regarding detection methods for audio and video deepfakes. Section 3 explains the details of the tackled problem and the proposed methods to fuse the audio and visual modalities. Section 4 describes the experimental setup used to validate the presented system, including details on the considered datasets. Section 5 collects all the achieved results providing detailed comments. Finally, Section 6 concludes the paper and outlines possible future works.

## 2. Deepfake Detection

In this section we introduce the reader to the deepfake detection task, providing a literature overview for the visual-only, audio-only and audio-visual deepfake detection scenarios.

### 2.1. Visual-Only Deepfake Detection

The rising of deepfake generation methods has posed a growing threat, leading to the development of numerous techniques to detect counterfeit videos and mitigate the damage they can cause. Generally, detection techniques leveraging visual content can be grouped into two categories, based on the approach they consider. The first group relies on manually-crafted features, while the second makes use of deep learning-based features.

Early forgery detection methods primarily depend on handcrafted features such as facial landmarks [12,13,14], optical flow [15] and various digital image processing techniques designed to enhance the visibility of artifacts [16].

With the advancement of video deepfake generation techniques and the higher quality of produced media, detecting deepfake video frames is becoming increasingly challenging using standard methods. Consequently, researchers have begun applying Deep Neural Networks (DNNs) with powerful feature extraction capabilities, aiming for more accurate and reliable detection processes with implicit feature learning.

As an example, the authors of [17,18] are pioneers in using DNNs to extract deep features from video frames. In [19] Convolutional Neural Networks (CNNs) and Long Short-Term Memory (LSTM) models are combined to detect deepfake videos generated using face-swapping techniques. The authors of [20] consider an ensemble of CNNs to detect video face manipulations, while those of [21] introduce the multi-head attention and fine-grained classification to detect deepfake videos, showing that the approach is robust to low-quality videos. Liu et al. [22] analyze the frequency domain signal of the deepfake videos and utilize the phase spectrum to obtain more information. Finally, the authors of [23] provide a semantic approach to deepfake detection, making use of a biological signal called Photoplethysmography (PPG), an optical technique that can detect subtle changes resulting in skin color due to blood in peripheral circulation through the face.

### 2.2. Audio-Only Deepfake Detection

The rapidly improving quality of synthetic speech generation has garnered increasing interest in speech deepfake detection. To do so, the scientific community has proposed numerous speech deepfake detectors that employ different detection approaches and strategies [24]. These can be broadly categorized into two groups based on the aspect they use to perform the detection task. The first group focuses on low-level features, looking for artifacts introduced by the generators at the signal level. The second group focuses on higher-level features representing more complex aspects as the semantic ones.

An example of an artifacts-based approach is presented in [25], where channel pattern noise analysis is used to secure Automatic Speaker Verification (ASV) systems against physical attacks. The authors of [26,27] exploit bicoherence features based on the assumption that a genuine recording has more significant non-linearity than a fake one. Alternatively, the authors of [28] propose an end-to-end network training for extracting deep features from speech, while those of [29] use Mel-Frequency Cepstral Coefficient (MFCC) features and an Support Vector Machines (SVM) classifier. Finally, new approaches to improve the practicality of existing detectors in real-world scenarios are proposed in [30,31].

Detection approaches that rely on semantic features operate under the assumption that, while deepfake generators can synthesize low-level aspects of the signals, they are unable to replicate more intricate high-level features. For instance, [32] exploits classic audio features inherited from the Music Information Retrieval (MIR) community to perform speech deepfake detection. Similarly, the authors of [33] leverage the lack of emotional content in synthetic voices generated via Text-to-Speech (TTS) techniques to recognize them, while [34] combines ASV and prosody features.

Other semantic aspects that can be exploited to perform speech deepfake detection are those related to the speaker identification problem, which refers to automatically identifying the identity of the speaker from a set of recognized voices [35]. At present, the most cutting-edge methods proposed to address this task are based on the use of x-vectors [36]. These are fixed-length features extracted by a DNN trained to discriminate between different speakers and can capture subtle speaker’s distinctive attributes, such as pronunciation, accent, and speaking style.

### 2.3. Audio-Visual Deepfake Detection

In recent years, there has been an increasing interest in the development of multimodal deepfake detection methods that can simultaneously analyze multiple modalities to achieve accurate and robust results. By analyzing multiple modalities at the same time, a detector can leverage inconsistencies or artifacts across different modalities, enhancing its detection capabilities. For instance, a deepfake video sequence may have realistic facial expressions but unnatural background sounds or mismatched lip movements.

For example, Ref. [37] leverages the incongruity between emotional cues portrayed by audio and visual modalities, while Ref. [11] integrates temporal data from image sequences, audio and video frames. Moreover, the results of [38] show that an ensemble of audio and visual baselines outperforms monomodal counterparts. The authors of [39] replace the standard MFCC features with an embedding of a DNN trained for automatic speech recognition, and then incorporate mouth landmarks. In [40], the authors establish a mapping between audio and video frames by analyzing the changes in the lip opening degree. In [7], the authenticity of a speaker is verified by detecting anomalous correspondences between his facial movements and what he says, while Ref. [41] exploits the inconsistency of lip shape between the audio and video signals.

Although multimodal detectors have shown great effectiveness, these systems are usually data-driven and require a large amount of data to be trained effectively. Unfortunately, in the literature there is a lack of challenging datasets that contain both fake video and audio, which makes it difficult to train and evaluate the performance of multimodal forensic detectors. In the recent years, few multimodal datasets have been proposed, containing both counterfeited video and audio tracks. These are DFDC [42], FakeAVCeleb [43], and DeepfakeTIMIT [44] with TIMIT-TTS [6]. In the following sections, we provide further details on these datasets and test our proposed multimodal detector on them.

## 3. Problem Formulation and Proposed Methodology

In this paper, we consider the problem of multimodal video deepfake detection and investigate whether this can lead to more robust and reliable predictions with respect to monomodal analyses. Given a video sequence depicting a front-facing person speaking, we aim at determining if the content is authentic or it has been synthetically generated or modified.

We tackle the task by considering a multimodal approach, meaning that we analyze both the person’s face and speech to perform the final prediction. In particular, we consider a video as fake when at least one between the visual and audio components is modified, while as real when both are authentic. In the following, we formulate the tackled problem in detail and illustrate the proposed methodology.

### 3.1. Problem Formulation

The problem we address can be formally defined as follows. Let us consider a video sequence under analysis $x_{\mathrm{AV}}$. We split it into two components: the time-series $x_{V}$ representing the temporal evolution of video frames showing the person’s face, and the time-series $x_{A}$ representing the temporal evolution of the audio track capturing the person’s speech.

Each of the two tracks $x_{V}$ and $x_{A}$ belong to a class $y_{V},y_{A}\in \left\{0,1\right\}$, where 0 means the signal of that modality is authentic while 1 indicates that it has been synthetically generated or edited. The class $y_{\mathrm{AV}}$ of the complete signal $x_{\mathrm{AV}}$ is defined as $y_{\mathrm{AV}}=y_{V}\vee y_{A}$, where ∨ is the logical “or” operator, meaning that we consider the complete signal as fake when at least one of its two modalities is fake.

Our goal is to develop a deepfake detector D that estimates the class of the original signal $x_{\mathrm{AV}}$. Given the video sequence $x_{\mathrm{AV}}$, the detector returns a real score ${\hat{y}}_{\mathrm{AV}}\in \left[0,1\right]$ which indicates the likelihood that $x_{\mathrm{AV}}$ is fake.

### 3.2. Proposed Methodology

**Figure 1:**
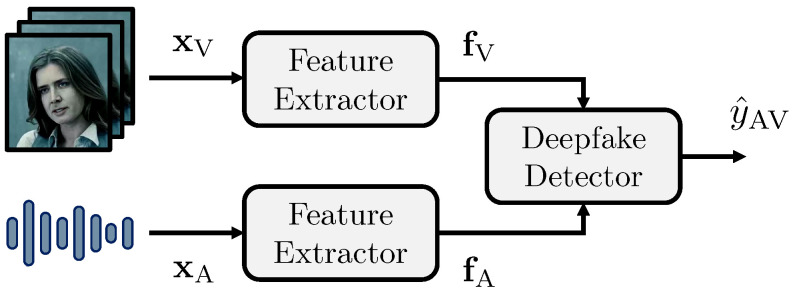
Proposed pipeline for multimodal deepfake detection.

Our proposed method is composed of two stages, as shown in Figure 1. In the first stage, we leverage state-of-the-art models to extract a collection of features from a subject’s facial and speech characteristics. In the second stage, we fuse these features to perform multimodal deepfake detection. In particular, we extract a feature set from some time instants of the input video, obtaining a temporal representation of it. Then, we exploit the temporal properties of the features using time-aware models to perform deepfake detection by fusing the two modalities, increasing the final detection accuracy.

In more details, we feed the signals $x_{V}$ and $x_{A}$ to two feature extractors $F_{V}$ and $F_{A}$, tailored to the visual and audio modalities respectively. The outputs of the two extractors are two sets of feature vectors

(1) $$f_{V}=F_{V}\left(x_{V}\right)\;\mathrm{and}\;f_{A}=F_{A}\left(x_{A}\right),$$

where each vector is extracted for a few time instants of the input signal.

We develop a deepfake detector D that takes as input the two sets of features $f_{V}$, $f_{A}$ and estimates a score ${\hat{y}}_{\mathrm{AV}}\in \left[0,1\right]$ related to the signal $x_{\mathrm{AV}}$. We define the estimated score as

(2) $${\hat{y}}_{\mathrm{AV}}=D\left(f_{V},f_{A}\right).$$

We consider different versions of the detector D, depending on the strategy we choose to perform the fusion between the two modalities.

#### 3.2.1. Feature Extraction

The feature extractors $F_{V}$ and $F_{A}$ we consider to compute the feature sets $f_{V}$ and $f_{A}$ are based on two well-established architectures proposed in the literature.

Regarding the visual modality, we exploit the EfficientNetB4 [45] network modified following the implementation proposed in [20], which investigates the ensembling of differently trained CNNs making use of attention layers and siamese training. The authors of the paper use the models’ ensemble to perform video deepfake detection, while we propose to use it as a feature extractor. To extract features from the video frames, we select the pixel area associated with the face of the person, then we pass the face-related frames to the models’ ensemble. We apply the exact implementation proposed in the original paper, therefore we refer the reader to that for more information. We decided to adopt this model as it has been shown to have excellent deepfake detection capabilities, which we believe can lead to adequate performance for the proposed multimodal classifier.

For the audio modality, we consider a Time-Delay Neural Network (TDNN) model coupled with statistical pooling to extract x-vector features from the input speech track. To do so, we exploit the pre-trained implementation provided by SpeechBrain [46]. The original task for which the model was proposed is speaker recognition. Here we use it as an embedding extractor, computing a feature vector for each time window of the audio signal under analysis.

It is worth noticing that, contrarily to $F_{V}$, $F_{A}$ is trained for a different task than the one at hand, i.e., deepfake detection. We do so because we want to adopt a semantic approach similar to the one used in [33,34], which has proved very effective against the detection of synthetic speech tracks. We face the deepfake detection by analyzing a set of high-level features, specifically related to the speaker’s identity, which we assume contain sufficient information to tackle also the considered task. Our rationale is that synthetic speech generators are very good at replicating low-level aspects of speech but fail to reproduce the most complex ones, such as the speaker’s identity. For this reason, we believe that high-level information can be exploited to discriminate between real and fake tracks.

The size of the feature sets $f_{V}$ and $f_{A}$ is equal to $N\times M_{V}$ and $N\times M_{A}$ respectively, where $M_{V}$ and $M_{A}$ are the lengths of the feature vectors extracted for each time instant while *N* is the numbers of time instants considered. In particular, since we want to provide an audio-visual representation of the input video sequence that is time-consistent between the two modalities, we extract the feature vectors for equally spaced time instants so that $f_{V}$ and $f_{A}$ are defined over the same number of time frames *N*.

#### 3.2.2. Deepfake Detection

The second part of the proposed pipeline consists of a binary classifier that takes as input the two feature sets $f_{V}$ and $f_{A}$ and returns a real score ${\hat{y}}_{\mathrm{AV}}$ associated with the input signal $x_{\mathrm{AV}}$. Since the features are defined as a function of the time instants, we implement the classifier using a time-aware model to exploit as much as possible the temporal correlations between and within the two modalities.

Specifically, we propose three different types of deepfake detectors D which differ in how the fusion between the feature sets $f_{V}$ and $f_{A}$ is performed. To better investigate the differences between the considered fusion strategies, we build the detectors D making use of the same inner network structure as a classifier to process the input feature sets. Since we work with different data modalities, we call the generic classifier model $C_{m}$, where m∈{V,A,AV} depending on the modality of the content analyzed, i.e., visual-only, audio-only and audio-visual.

**Figure 2:**
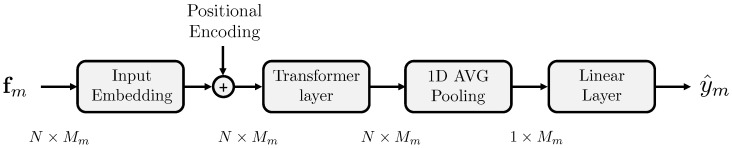
Architecture of the classifier $C_{m}$.

The proposed architecture for $C_{m}$ consists of a Transformer-based model [47] that leverages the temporal aspect of the features. It comprises an input embedding layer that maps the input features to a hidden dimension, a positional encoding layer, a transformer encoder layer that processes the input sequence, and a fully connected layer that performs the final binary classification. The output layer employs a softmax function to return a probability estimate of whether the analyzed input feature is extracted from a fake signal. The dimensionality of the latent space at the output of the transformer is the same as that of its input. This is because this approach enables the model to better preserve and analyze the information contained within the input sequence. Figure 2 shows the generic architecture of the proposed model $C_{m}$. The size $M_{m}$ of the input feature vector varies according to the considered modality *m*.

**Figure 3:**
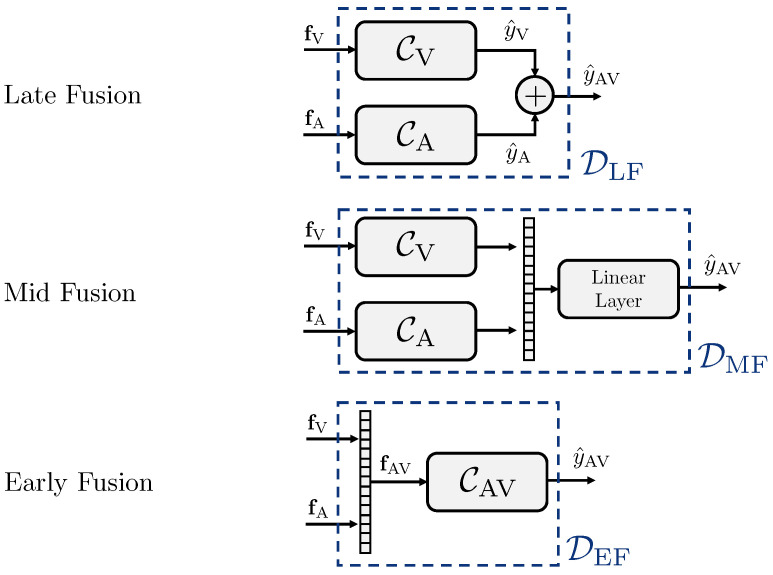
Different fusion strategies considered to perform multimodal deepfake detection.

In the next lines, we list the three fusion strategies we propose in this work. These offer practical approaches for performing multimodal deepfake detection, focusing on efficient implementations and usability in real-world scenarios. The proposed setups can be readily implemented on existing monomodal deepfake detectors or serve as the foundation for building new models, depending on the needed requirements and preferences. For clarity’s sake, Figure 3 shows the pipelines of the strategies, called *Late Fusion*, *Mid Fusion* and *Early Fusion*.

***Late Fusion.*** In the *Late Fusion* strategy, the deepfake detector considers a dedicated classifier for each modality, which we call $C_{V}$ and $C_{A}$. We separately train the two classifiers only on visual ($C_{V}$) and audio ($C_{A}$) data. In testing phase, every classifier takes as input the feature set associated with the related modality and returns a score such that

(3) $${\hat{y}}_{V}=C_{V}\left(f_{V}\right)\;\mathrm{and}\;{\hat{y}}_{A}=C_{A}\left(f_{A}\right).$$

The final multimodal score assigned to the video sequence is computed by averaging the monomodal ones,

(4) $${\hat{y}}_{\mathrm{AV}}=\left({\hat{y}}_{V}+{\hat{y}}_{A}\right)/2.$$

We define this detector as $D_{\mathrm{LF}}$, being

(5) $${\hat{y}}_{\mathrm{AV}}=D_{\mathrm{LF}}\left(f_{V},f_{A}\right)=\left(C_{V}\left(f_{V}\right)+C_{A}\left(f_{A}\right)\right)/2.$$

***Mid Fusion.*** Regarding the *Mid Fusion* strategy, we consider two classifiers $C_{V}$ and $C_{A}$ that are still separated for the two modalities, though being merged in their final dense layers. In more details, for each classifier, we extract the feature embedding obtained before the final fully connected layer. We concatenate the embeddings associated with each data modality, ending up with a multimodal embedding vector with size $1\times (M_{V}+M_{A})$. Then, we provide the computed multimodal embedding as input to a fully-connected layer that returns the final score ${\hat{y}}_{\mathrm{AV}}$.

Differently from the *Late Fusion* strategy, we train the *Mid Fusion* strategy end-to-end. In this way, the two classifiers update their related parameters considering the contributions of both modalities. We define the *Mid Fusion* detector as $D_{\mathrm{MF}}$, being

(6) $${\hat{y}}_{\mathrm{AV}}=D_{\mathrm{MF}}\left(f_{V},f_{A}\right).$$

***Early Fusion.*** In the *Early Fusion* strategy, we consider a unique classifier $C_{\mathrm{AV}}$ that takes as input the concatenation of the two feature sets $f_{\mathrm{AV}}=\left[f_{V},f_{A}\right]$ and directly returns the score ${\hat{y}}_{\mathrm{AV}}$. The feature vectors of the two modalities are concatenated along the feature-dimension, so that the final size of $f_{\mathrm{AV}}$ is equal to $N\times M_{\mathrm{AV}}$, where $M_{\mathrm{AV}}=M_{V}+M_{A}$.

The idea behind this fusion strategy is that, when we provide the detector with multimodal information at an early stage, it can exploit the audio-visual correlations better, which may benefit the final detection capabilities. We define the *Early Fusion* detector as $D_{\mathrm{EF}}$, being

(7) $${\hat{y}}_{\mathrm{AV}}=D_{\mathrm{EF}}\left(f_{V},f_{A}\right)=C_{\mathrm{AV}}\left(f_{\mathrm{AV}}\right).$$

## 4. Experimental Setup

In this section we provide the reader with some insights regarding the experimental setup used to assess the performances of the proposed detectors. First, we describe the datasets considered for training and testing all the stages of the systems. Then, we give more details on the processing pipeline, providing the parameters for the extraction of audio and visual features and those for the deepfake detector. Finally, we present the procedure used to train the considered models.

### 4.1. Considered Datasets

As mentioned in Section 2, in the multimedia forensics literature the multimodal deepfake datasets that have been released are few and are not enough to perform comprehensive studies by training models on specific sets and testing them on unseen data. This is a significant limitation that restricts the development of new multimodal detectors. In this paper, we try to overcome this problem and show how multimodal analyzes can be more robust and reliable even when the considered models are trained on monomodal datasets that are unrelated to each other. Following this approach, we train the proposed detectors on visual-only (i.e., FaceForensics++) or audio-only (i.e., ASVspoof 2019) monomodal deepfake datasets and test them on multimodal audio-video corpora. Here we present in detail all the considered datasets.

#### 4.1.1. Training Datasets

***FaceForensics++*** **[18].** This is a visual-only deepfake dataset containing 5000 videos which were generated using four different deepfake generation methods using a base set of 1000 real YouTube videos. It includes two partitions corresponding to different compression pipelines applied to the videos. In particular, the dataset includes two values of Quantization Parameter (QP), QP = 23 and QP = 40, where higher QP means lower quality.

We use this dataset to train the $C_{V}$ model, considering the *train* and *validation* splits released by the authors. Then, we exploit the *test* split for a preliminary monomodal evaluation. As for the two partitions of QP, we merge them to make the training and evaluation processes more robust.

***ASVspoof 2019*** **[48].** This is a speech audio dataset that contains both real and synthetic tracks generated based on the VCTK corpus [49]. In particular, we consider the Logical Access (LA) partition, which relates to the synthetic speech detection problem. This contains more than 120,000 audio tracks, all at a sampling frequency of $f_{s}$ = 16 kHz. The LA partition is split into three sub-partitions, namely *train*, *dev* and *eval*, which contain authentic signals along with synthetic speech samples generated with various methods. The *train* and *dev* partitions have been created using a set of six synthesis algorithms, while *eval* includes samples generated with thirteen techniques, different from those used in *train* and *dev*.

We use the *train* and *dev* partitions during the training phase of the $C_{A}$ model, while we exploit the *eval* split to test the detector in a monomodal scenario.

#### 4.1.2. Evaluation Datasets

We evaluate the proposed audio-video detectors on multiple state-of-the-art multimodal deepfake datasets. We do so since we want to test their robustness against various types of forgeries and anti-forensic attacks, aiming at replicating real-world evaluation scenarios. In the forensic field, it is crucial for a detector to exhibit reliable and robust predictions even when tested on data that differs from the ones seen during training. Hence, the ability of a model to generalize across different types of data becomes an important aspect to consider and by testing it on diverse datasets we can effectively evaluate their performance in these terms. Here we introduce the deepfake datasets we considered in the multimodal evaluation setup.

***FakeAVCeleb*** **[43].** This is a multimodal deepfake dataset that contains 500 real videos extracted from the VoxCeleb2 corpus [50], used as a base set to generate around 20,000 deepfake videos through various deepfake generation methods. Deepfake video frames have been generated with Faceswap [51] and FSGAN [52], while the deepfake audios have been synthesized using Real-Time Voice Cloning (RTVC) [53]. Then, Wav2Lip [54] has been applied to synchronize the video frames with the audio.

***DFDC*** **[42].** This multimodal deepfake dataset contains nearly 120,000 videos, of which 100,000 are labeled as “Fake” and the rest as “Real”. The videos are divided into 50 folders, numbered from 0 to 49, where each subset contains a set of real videos, along with all derivative fake videos. While the videos are largely visual-only fakes, some samples included in divisions 45 to 49 contain falsified audio in addition to possible falsified video. Since our goal is to perform multimodal experiments, we consider only the videos within these folders as test dataset, for a total of 12,547 samples.

***VidTIMIT*** **[55].** This is a multimodal dataset that includes only real video recordings of 43 people reciting short sentences, considering 10 videos per subject, for a total of 430 videos. It has been widely used for research on topics such as automatic lip reading, multi-view face recognition, multi-modal speech recognition and person identification. The recorded sentences are extracted from the test section of the TIMIT corpus [56].

***DeepfakeTIMIT*** **[44].** This is a video deepfake dataset including only fake video samples, generated starting from the VidTIMIT corpus presented above. The forgery process regards only the visual content of the video sequences; specifically, the forged video frames were generated with a Generative Adversarial Network (GAN)-based approach developed from Faceswap [51]. The generated deepfakes belong to 32 subjects and are released in two versions: a low quality (LQ) and a high quality (HQ), with different frame sizes. This set includes a total of 640 videos with swapped faces (320 for each quality version). In our experiments, we merge LQ and HQ subsets, considering them as a unique corpus.

***TIMIT-TTS*** **[6].** This is a speech dataset including only fake audio samples, generated starting from the VidTIMIT corpus. This dataset contains four partitions, corresponding to different post-processing pipelines applied to audio tracks. Here we consider the Dynamic Time Warping (DTW) subset, which includes almost 20,000 synthetic speech tracks synthesized using twelve different TTS algorithms and then passed through a DTW system to sync them to the reference videos, increasing their realism. This corpus can be used as a standalone synthetic audio dataset or combined with VidTIMIT and DeepfakeTIMIT sets to perform multimodal research.

In the following experiments, we combine the VidTIMIT, DeepfakeTIMIT and TIMIT-TTS datasets and consider them as a unique multimodal deepfake corpus, which we refer to as TIMIT.

### 4.2. Processing Pipeline

#### 4.2.1. Feature Extraction

The two feature extractors $F_{V}$ and $F_{A}$ work to capture the content of the input video sequence over time. In particular, to capture fine-grained temporal changes, we consider an extraction frequency equal to 10 Hz. Concerning visual information, this is done by selecting 10 evenly spaced frames within a second and extracting a feature from each of them. Concerning speech information, we divide the signal considering non-overlapped time windows of 100 ms and extracting a feature from each of them. At the end of the feature extraction process, visual and spatial features are synchronized and describe information evolving in time at 10 samples per second. Regarding the temporal dimension, we analyze the input signals over a time window $T_{W}=3.0\;s$. We adopt this window length because, from preliminary experiments, it turned out to be a good compromise between the shortness of the window and the performance of the detector, which is desired in a real-world scenario.

For both feature extractors, we exploit the pre-trained models released by the authors of the respective papers. In particular, $F_{V}$ was trained on FaceForensics++, while $F_{A}$ was trained on Voxceleb [57] and Voxceleb2 [50] datasets, considering audio data sampled at 16 kHz. Finally, at each considered time instant, the number of features extracted from the visual content is equal to $M_{V}=1072$, while those extracted from the audio content are $M_{A}=512$. Considering 10 samples per second over a time window of 3.0 s, the final temporal dimension of the features is equal to N=30. Therefore, the size of the visual feature $f_{V}$ is equal to 30×1072, while the size of the audio feature $f_{A}$ is equal to 30×512.

#### 4.2.2. Deepfake Detector

As reported in Section 3, all the considered deepfake detectors share the same architecture $C_{m}$. The input shape of the networks is equal to $N\times M_{m}$, where $M_{m}$ depends on the feature set we are considering, i.e., m∈{V,A,AV}. All the considered models contain a transformer encoder that presents a single hidden layer with 8 attention heads, 0.1 dropout, and GELU as activation function.

Each input feature set is normalized to have zero mean and unitary variance, both in training and test. In the *Early Fusion* strategy, when the features are concatenated before feeding them to the model, the normalization is performed independently between the modalities, prior to the concatenation.

### 4.3. Training Strategy

All the hyperparameters used to train the considered models have been selected to maximize the classification accuracy. In particular, we consider a number of epochs equal to 150 with an early stopping patience at 15 epochs, weighted cross-entropy as loss function and Adam optimization. We adopt a learning rate equal to e-3, a weight decay of e-4, and we reduce the learning rate on plateau of the validation loss by a factor 0.1.

During training we pay attention to balancing the classes in order to compensate for the imbalance of the training datasets. In particular, we oversample the tracks of the less represented class, ensuring that each training batch contains the same number of samples from the “Real” and “Fake” classes.

## 5. Results

In this section we analyze and discuss the results achieved by the proposed techniques for multimodal deepfake detection.

### 5.1. Evaluation Metrics

We evaluate the performances of the considered detectors using Receiver Operating Characteristic (ROC) curves and confusion matrices, considering as evaluation metrics the Area Under the Curve (AUC) and the Balanced Accuracy (BA). In general, we evaluate the BA as a function of the threshold *t* applied to the likelihood score returned by the detector to estimate the class of the query video sequence (i.e., “Real” or “Fake”). If the likelihood exceeds the threshold, the sequence is classified as “Fake”, otherwise it is classified as “Real”. We define the BA at threshold *t* as

(8) $${\mathrm{BA}}_{t}=\frac{{\mathrm{TPR}}_{t}+{\mathrm{TNR}}_{t}}{2},$$

where ${\mathrm{TPR}}_{t}$ and ${\mathrm{TNR}}_{t}$ are the True Positive Rate (TPR) and True Negative Rate (TNR) of the tackled binary decision problem at fixed threshold *t*, respectively. Optimal performances are achieved when both AUC and BA approach values next to 1. In all the considered investigations, we apply a standard threshold t=0.5 to the output likelihood, ending up with ${\mathrm{BA}}_{0.5}$ as evaluation metrics. Nonetheless, we show that there are a few scenarios where better results can be achieved by aptly modifying this value.

### 5.2. Monomodal Results

As a preliminary experiment, we test the effectiveness of the monomodal detectors in their respective domains. The reason behind this choice is that good visual and audio classifiers are essential for building an excellent multimodal detector. Our proposal focuses on fusion strategies designed for merging monomodal deepfake detectors. As a result, the performances of the fused model are directly influenced by those of the starting detectors being used. If the monomodal detectors do not work properly, it would be necessary to act on them before their fusion in the multimodal investigations. Therefore, we exploit the monomodal scores defined in (3) to evaluate our performances on the test partitions of monomodal datasets (i.e., FaceForensic++ for visual data and ASVspoof 2019 for audio data).

**Figure 4:**
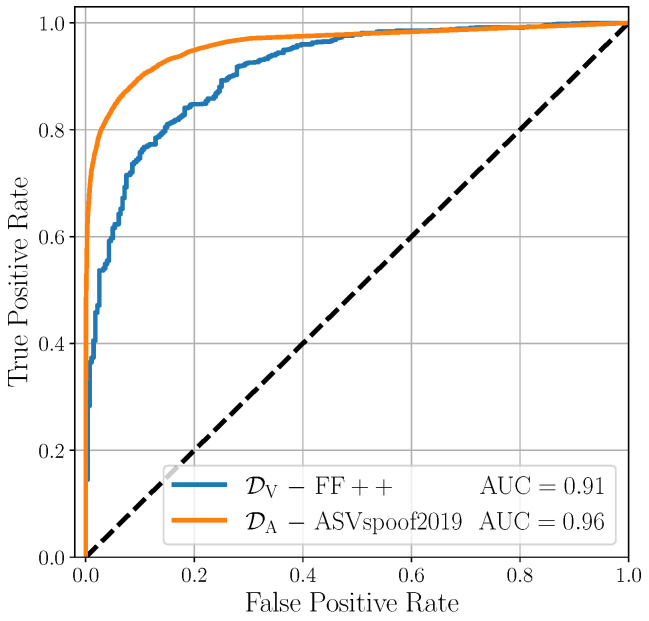
Evaluation of the considered detectors on monomodal datasets.

Figure 4 shows the results of this preliminary analysis. The two classifiers show excellent detection performances, with an AUC of 0.91 for $D_{V}$ and an AUC of 0.96 for $D_{A}$, along with ${\mathrm{BA}}_{0.5}$ of 0.83 and 0.90, respectively. These results are consistent with those of many cutting-edge detectors reported in the literature [20,34], indicating that the proposed monomodal classifiers are suitable for subsequent multimodal experiments.

### 5.3. Multimodal Results

In each of the following multimodal experiments, we evaluate the proposed detectors only on datasets different from the monomodal datasets used to train the classifiers. Performing cross-dataset tests represents a challenging scenario that resembles “in-the-wild” conditions, which enables to evaluate the robustness of the proposed strategies against different forgeries and anti-forensic attacks. Also, we are aware that training on monomodal datasets could impact the achieved performance on multimodal ones. A notable limitation of this approach is that the detectors are unable to leverage all the intra-modality relationships within the content since these relationships are not accessible during training. Due to this aspect, the proposed system is unable to detect synthetic content that appears realistic in individual modalities but lacks synchronization between audio and video, even if simpler detectors trained explicitly with this purpose could easily spot such inconsistencies. Still, we want to investigate whether a modality fusion process can improve the detection capabilities even though the data seen in training are “partial”.

#### 5.3.1. Best Fusion Strategy Selection

As a first experiment, we examine the fusion strategies introduced in Section 3.2.2 and contrast their respective performances, investigating which one leads to more robust predictions. For this test we evaluate the detectors only on multimodal deepfakes that share the same class between video and audio (i.e., both are either real or fake), excluding videos where only one of the modalities is edited (e.g., fake video and real audio or vice versa).

**Figure 5:**
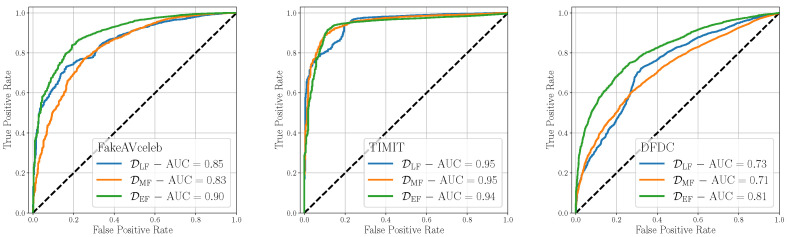
Evaluation of the considered detectors on multimodal datasets considering different fusion strategies.

Figure 5 shows the ROC results of this analysis, broken down by the considered test dataset. On average, *Early Fusion* is the most effective fusion strategy, enabling to achieve AUCs larger or equal 0.90 for two datasets out of three, and being the best performing strategy on the remaining dataset. As a matter of fact, *Early Fusion* can exceed the other fusion strategies by 7% and 10% on FakeAVceleb and DFDC datasets, respectively, while being competitive on the TIMIT dataset. We believe this technique enables the detector to deeply analyze both the relationships between and within the modalities, thereby enhancing the robustness of its predictions. We observe that the scored AUC values display significant variations depending on the test dataset under analysis, reaching poor values in the case of the DFDC set. This is likely due to distinct characteristics between training and test data, which can adversely impact the detector predictions.

One further approach we could consider is the recursive application of a *Late Fusion* strategy, fusing the scores obtained from the three proposed methods by averaging them. The results achieved using this strategy are AUC = 0.88 for FakeAVceleb, AUC = 0.96 for TIMIT, and AUC = 0.78 for DFDC. While we acknowledge that on certain datasets this approach improves the results reported before, we believe that it brings limited novelty to the analysis. First, it considers a fusion strategy that has already been previously explored. Additionally, from a computational perspective, this strategy may not be practical as it needs to use three different models to obtain a score. This can introduce unnecessary computational overhead without significantly enhancing the overall performance. Consequently, for these reasons, we decided not to consider this approach in the following analyses.

**Figure 6:**
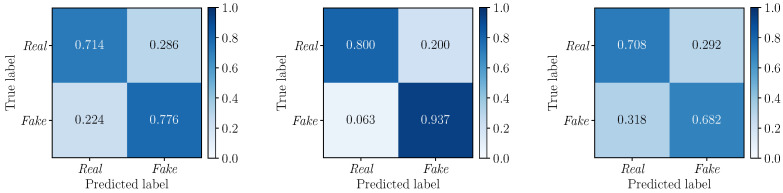
Confusion matrices obtained by considering the $D_{\mathrm{LF}}$ detector (*Late Fusion*) on the FakeAVceleb (**left**), TIMIT (**center**) and DFDC (**right**) datasets. The corresponding ${\mathrm{BA}}_{0.5}$ values are 0.75, 0.87, and 0.69, respectively.

**Figure 7:**
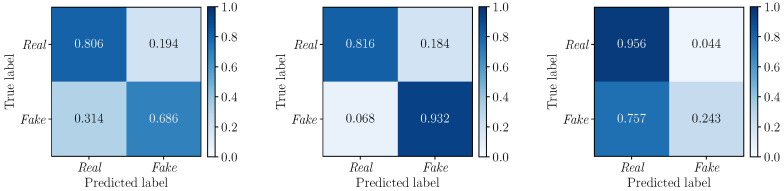
Confusion matrices obtained by considering the $D_{\mathrm{MF}}$ detector (*Mid Fusion*) on the FakeAVceleb (**left**), TIMIT (**center**) and DFDC (**right**) datasets. The corresponding ${\mathrm{BA}}_{0.5}$ values are 0.75, 0.87, and 0.70, respectively.

**Figure 8:**
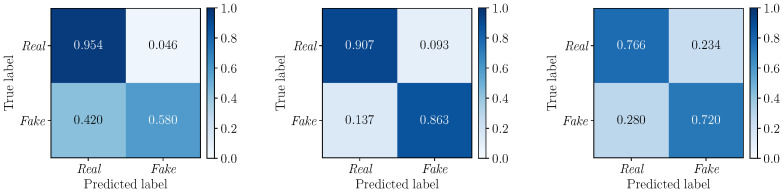
Confusion matrices obtained by considering the $D_{\mathrm{EF}}$ detector (*Early Fusion*) on the FakeAVceleb (**left**), TIMIT (**center**) and DFDC (**right**) datasets. The corresponding ${\mathrm{BA}}_{0.5}$ values are 0.77, 0.88, and 0.74, respectively.

To further deepen our investigations, we compute the confusion matrices to evaluate the performance of the detectors $D_{\mathrm{LF}}$, $D_{\mathrm{MF}}$ and $D_{\mathrm{EF}}$ on the three considered multimodal deepfake datasets. Results are depicted in Figure 6 ($D_{\mathrm{LF}}$), Figure 7 ($D_{\mathrm{MF}}$) and Figure 8 ($D_{\mathrm{EF}}$). In all cases, we apply a standard fixed threshold t=0.5 to the estimated likelihood associated with each video sequence. The ${\mathrm{BA}}_{0.5}$ values reinforce the results observed with the ROC curves, with *Early Fusion* that proves again to be the best fusion strategy. However, while on TIMIT and DFDC datasets this strategy reports well balanced TPR and TNR with similar values, *Early Fusion* results on FakeAVceleb are strongly unbalanced towards the “Real” class (i.e., ${\mathrm{TPR}}_{0.5}=0.954$ vs. ${\mathrm{TNR}}_{0.5}=0.580$).

Motivated by this observation, we extend our investigations, computing the BA by varying the threshold applied to the scores. Then, for each of the tackled experiments, we consider the optimal threshold value *t* determined to maximize the BA value. Notice that this experiment enables to test the robustness of the proposed detectors when dealing with unseen data. If the maximum achieved BA (by varying all the possible thresholds) shows similar to the ${\mathrm{BA}}_{0.5}$ and reports acceptable values, this means the detector is well calibrated and it is robust to unknown data.

Table 1 compares the BA values at different thresholds for all the considered cases. For the sake of clarity, we also include the achieved AUCs. The results show that using a fixed threshold t=0.5 does not particularly affect the results obtained, with the BA values not considerably changing between the two considered scenarios. FakeAVceleb is the dataset showing the most notable changes, with a 5% accuracy improvement when considering the best threshold for the detector $D_{\mathrm{EF}}$. However, this compromise is acceptable given the other accomplished results. In general the scores indicate the robustness of the proposed detectors, which are capable of handling unseen data.

Since *Early Fusion* proves to be the best-performing strategy among the three proposed ones, we consider this for all the remaining evaluations.

#### 5.3.2. Multimodal vs. Monomodal Detection

We now compare the performances of the developed *Early Fusion* multimodal detector with those of the corresponding monomodal models. We do so since we want to test whether a multimodal analysis is more robust and reliable than a monomodal one. We recall again that our multimodal models are trained solely on monomodal data, so they do not require any additional training datasets. In this experiment the monomodal models serve as a baseline for our study. The purpose is to assess whether the multimodal approach proposed in our work offers advantages compared to a monomodal one. By comparing the performance of the proposed detector against the baselines, we can determine the potential benefits and improvements achieved through a multimodal approach. As done in the previous experiment, we only analyze deepfakes in which both the video and audio signals belong to the same class and exclude samples where only one modality is manipulated. This is done because monomodal detectors, by nature, cannot detect these types of forgeries.

**Figure 9:**
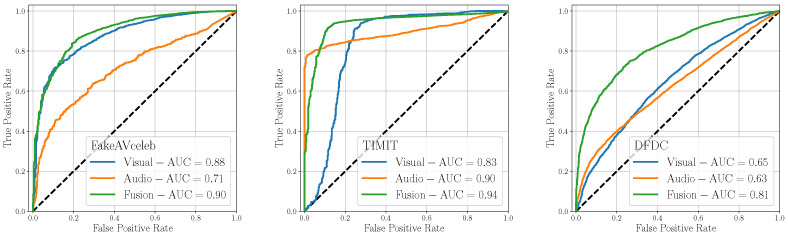
Evaluation of the considered detectors on multimodal datasets considering monomodal (i.e., visual-only or audio-only) against multimodal approaches.

Figure 9 shows the ROC results broken down for each test dataset, while Table 2 compares the AUC, BA and ${\mathrm{BA}}_{\mathrm{best}t}$ values for the three methods. The multimodal approach consistently outperforms the monomodal detectors, supporting the considerations made in our investigations.

#### 5.3.3. Mixed Class Experiments

As a last experiment, we expand the analysis to include also deepfakes with mixed class labels (i.e., real video frames and fake audio or vice versa). In doing so, we evaluate how much the performances of the detectors are affected when dealing with mixed classes and we determine if they can effectively address this challenging task.

To fully understand the discrimination capabilities of the detectors, we exclude the simplest case from the test samples, corresponding to the one in which both audio and video frames are modified. Indeed, we have already evaluated the detection performance of the proposed classifiers on this type of data. We now want to investigate their ability in a more challenging scenario, i.e., identifying samples where only one between audio and visual modalities is forged.

For this specific experiment, we expand our analysis and include additional training strategies. Up to this point, all detectors were trained only on data associated with the same class across modalities, i.e., $y_{V}=y_{A}$, to ensure consistency with the data used during the tests. Since here we also consider mixed classes, we train the detectors on mixed classes as well, i.e., when $y_{V}\neq y_{A}$, and compare their performances with those of the detectors trained only on consistent classes. As previously reported in Section 4.3, we keep the balance between the “Real” and “Fake” classes, but we also ensure that all cases falling into the “Fake” class, i.e., $\left[y_{V},y_{A}\right]=\left[1,0\right]$, $\left[y_{V},y_{A}\right]=\left[0,1\right]$ and $\left[y_{V},y_{A}\right]=\left[1,1\right]$, are equally represented.

**Figure 10:**
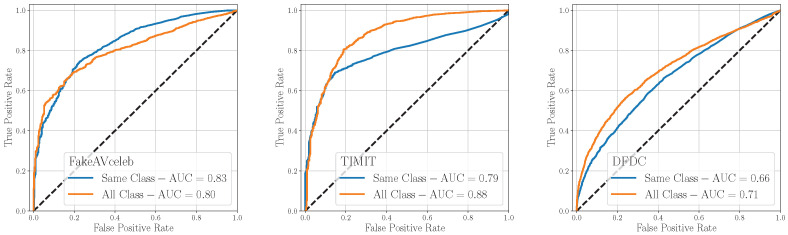
Evaluation of the $D_{\mathrm{EF}}$ detector (*Early Fusion*) on mixed classes (real audio and fake video and viceversa). The case where both video and audio are fake is excluded.

Figure 10 shows the ROC results, where the original training strategy has been indicated as *Same Class* while the other as *All Class*, meaning that it includes both the scenarios in which $y_{V}=y_{A}$ (same class) and $y_{V}\neq y_{A}$ (mixed class). The $D_{\mathrm{EF}}$ model achieves the best average results when trained following the *All Class* strategy (average AUC = 0.80 vs. average AUC = 0.76 of the *Same Class*). Training the detector following the *All Class* strategy enables to learn additional traces within modalities, which can benefit the discrimination of mixed-classes deepfakes. The use of intra-modal information is noteworthy because it enhances the detection accuracy of the system, even when it is trained on single-modal data, as in our case. We are confident that training the model on a multimodal deepfake dataset would further improve its performance, as it would increase the number of intra-modal traces. These outcomes prove another time the robustness and effectiveness of the proposed approach, capable of reaching good performance on unseen data and different test datasets.

## 6. Conclusions and Future Works

In this paper we presented a novel approach for detecting multimodal deepfake videos by combining visual and audio information. The proposed method was used to determine the authenticity of an input video sequence, combining data-driven features extracted from the visual content with speaker-identity features from the audio stream. We evaluated several training and test methods, and various modality fusion strategies. The results indicate that robust predictions are achieved when an *Early Fusion* approach is considered.

The peculiarity of the proposed detector is that its training phase does not take place on multimodal deepfake data but on monomodal deepfake samples (i.e., that contain either modified video frames only or modified audio samples only), thus not requiring additional multimodal training data. Despite this “partial” training strategy, the model is able to outperform detectors trained only on monomodal data, underlining the goodness of using a multimodal approach.

In future studies we want to experiment with new methods of fusion between modalities, such as “informed” fusion methods. This means the contribution of the different modalities is weighted with respect to the relevance they may have in the accuracy of the final prediction.

## Figures and Tables

**Table 1:** AUC and BA values obtained testing the proposed detectors considering different fusion strategies and different thresholds *t*.

	FakeAVceleb			TIMIT			DFDC		
	*Late*	*Mid*	*Early*	*Late*	*Mid*	*Early*	*Late*	*Mid*	*Early*
AUC	0.85	0.83	0.90	0.95	0.95	0.94	0.73	0.71	0.81
${\mathrm{BA}}_{0.5}$	0.75	0.75	0.77	0.87	0.87	0.88	0.69	0.60	0.74
${\mathrm{BA}}_{\mathrm{best}t}$	0.78	0.76	0.82	0.87	0.90	0.90	0.70	0.67	0.74

**Table 2:** AUC and BA values at different thresholds *t*, obtained testing the proposed detectors on multimodal datasets considering monomodal (i.e., visual-only or audio-only) against $D_{\mathrm{EF}}$ (*Early Fusion*) detector.

	FakeAVceleb			TIMIT			DFDC		
	*Visual*	*Audio*	*Fusion*	*Visual*	*Audio*	*Fusion*	*Visual*	*Audio*	*Fusion*
AUC	0.88	0.71	0.90	0.83	0.90	0.94	0.64	0.74	0.81
${\mathrm{BA}}_{0.5}$	0.80	0.64	0.77	0.83	0.76	0.88	0.60	0.58	0.74
${\mathrm{BA}}_{\mathrm{best}t}$	0.80	0.67	0.82	0.83	0.88	0.90	0.61	0.60	0.74

## Data Availability

Not applicable.
